# Magnetization-transfer flow-independent dark-blood delayed enhancement cardiac MRI optimizes discrimination of ST-elevation myocardial infarct borders

**DOI:** 10.1007/s00330-024-11192-7

**Published:** 2024-12-05

**Authors:** Paulina Poskaite, Christian Kremser, Mathias Pamminger, Felix Troger, Gert Reiter, Sebastian J. Reinstadler, Bernhard Metzler, Wolfgang G. Rehwald, Raymond J. Kim, Agnes Mayr

**Affiliations:** 1 University Clinic of Radiology, Medical University of Innsbruck, A-6020 Innsbruck, Austria; 2 Research and Development, Siemens Healthcare Diagnostics GmbH, A-8054 Graz, Austria; 3 University Clinic of Internal Medicine III, Cardiology and Angiology, Medical University of Innsbruck, A-6020 Innsbruck, Austria; 4 Siemens Medical Solutions, Malvern, Pennsylvania US; 5 Duke Cardiovascular Magnetic Resonance Center, Duke University Medical Center, Durham, North Carolina US

**Keywords:** Magnetic resonance imaging, Cardiac imaging techniques, Myocardial infarction

## Abstract

**Objectives:**

To prospectively compare image quality and infarct sizing methods between magnetization-transfer “flow-independent dark-blood delayed enhancement” (MT-FIDDLE) and standard “bright-blood”-late gadolinium enhancement (LGE) cardiac-magnetic-resonance (CMR) sequence.

**Methods:**

“Bright-blood”-LGE and MT-FIDDLE sequence were acquired in 110 patients at 4 days (*n* = 33), 4 months (*n* = 39) and 12 months (*n* = 38) after acute ST-elevation myocardial infarction (STEMI). Subjective image quality, including confidence in infarct segmentation and blood-pool bordering, were each rated on a 4-point Likert scale. Objective image quality was assessed by the detectability index (DI). Infarct volumes derived via full-width at half-maximum (FWHM) and different number of standard deviations (“n-SD”) methods on MT-FIDDLE images were compared with FWHM and reference 5-SD results from “bright-blood-LGE images.

**Results:**

Overall subjective median image quality was excellent for both LGE sequences. Qualitative analysis revealed a significantly higher confidence in infarct segmentation and in blood-pool bordering for MT-FIDDLE as compared to “bright-blood”-LGE (all *p* < 0.001). Infarct volumes assessed by the FWHM technique on MT-FIDDLE and “bright-blood”-LGE showed excellent agreement overall (Concordance correlation coefficient, CCC = 0.96). The 3-SD technique for MT-FIDDLE showed the best agreement with the 5-SD method for “bright-blood”-LGE overall (CCC = 0.94), as well as in the subgroup with excellent confidence in infarct segmentation on “bright-blood”-LGE (CCC = 0.96). DI of scar versus LV blood-pool was higher for MT-FIDDLE (8.9 ± 5.5) compared to “bright-blood”-LGE sequence (2.0 ± 1.5; *p* < 0.001).

**Conclusion:**

MT-FIDDLE significantly optimizes the discrimination between myocardial infarction and adjacent blood-pool in STEMI patients. As compared to the established 5-SD technique on “bright-blood”-LGE, the 3-SD method on MT-FIDDLE results in consistent infarct volumes.

**Key Points:**

***Question*** *Does magnetization-transfer “flow-independent dark-blood delayed enhancement” (MT-FIDDLE) offer any benefits over standard “bright-blood”-late gadolinium enhancement (LGE) cardiac-magnetic-resonance (CMR) for identifying STEMI infarct borders?*

***Findings*** *MT-FIDDLE image quality was higher than LGE CMR and measured infarct volume comparability to the standard 5-SD-threshold-technique.*

***Clinical relevance*** *MT-FIDDLE facilitates the assessment of myocardial infarctions at the subendocardial border, improving the discrimination between myocardial infarction and adjacent blood-pool in STEMI patients.*

## Introduction

Cardiac magnetic resonance (CMR) late gadolinium enhancement (LGE) “bright-blood”-imaging is the reference standard for imaging of ischemic and non-ischemic replacement fibrosis [1, 2]. After ST-segment elevation myocardial infarction (STEMI), the assessment of infarct volume and transmurality is critical for the prognosis of major adverse cardiovascular events [3] and for clinical decision-making prior to revascularization [4–6]. Conventional “bright-blood”-LGE has proven to permit accurate evaluation of infarct extent with excellent agreement to histopathologically assessed infarct volumes [7]. However, as in “bright-blood”-LGE, both the infarcted myocardium and the blood-pool are of bright signal, the detection and delineation of the infarcted tissue at the subendocardial border may be limited. This specific restriction may particularly complicate the assessment of subendocardial or small myocardial infarctions, which have also been proven to impact prognosis [8].

To overcome this disadvantage of the “bright-blood”-LGE sequence, conventional “dark-blood” CMR sequences are of limited use because they usually depend on a long T1-value of blood as well as sufficient blood flow during the relaxation time and thus do not work after contrast administration (CA) [9]. For this reason, several special techniques for “dark-blood”-LGE have been suggested and evaluated [9–14]. One of these techniques, the recently introduced flow-independent dark-blood delayed enhancement sequence (FIDDLE), uses a phase-sensitive inversion recovery (PSIR) sequence together with a magnetization preparation module that affects the magnetizations of myocardium and blood differently. Utilizing a magnetization-transfer (MT) preparation scheme before the inversion pulse, MT-FIDDLE has proven a higher accuracy for diagnosis of myocardial infarction compared to the “bright-blood”-LGE sequence in an animal model of MI with pathology as the reference standard and a small cohort of patients [9]. Foley et al demonstrated a significantly higher scar-to-blood contrast-to-noise ratio (CNR) of a T1ρ-FIDDLE and a blood-nulled phase-sensitive inversion recovery (PSIR) compared to the standard LGE sequence in a prospective comparative study with 30 patients after myocardial infarction [15]. However, MT-FIDDLE has not yet been tested in a clinical setting on a large patient population with different stages of myocardial infarction (acute, subacute and chronic).

Thus, the present study aims were as follows: (1) to compare both MT-FIDDLE and “bright-blood”-LGE CMR sequences on a large patient cohort after STEMI regarding image quality; and (2) to determine the most appropriate method for quantification of infarct volume in MT-FIDDLE compared to “bright-blood”-LGE.

## Methods

### Study population

This study was conducted in accordance with the Declaration of Helsinki and approved by the local research ethics committee of the Medical University of Innsbruck. Written informed consent was obtained from all patients.

**Fig. 1:**
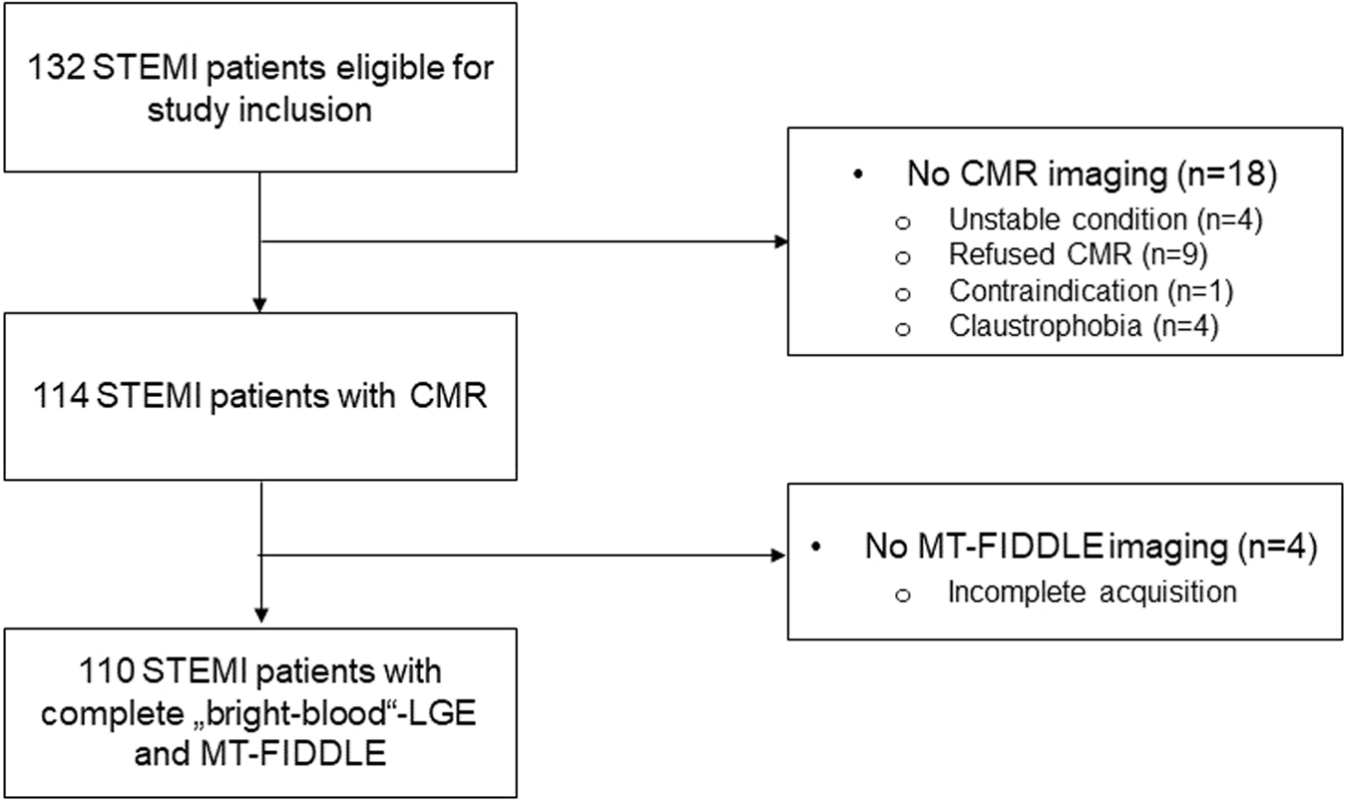
Flow chart of the present study. STEMI, ST-elevation myocardial infarction; CMR, cardiac magnetic resonance; LGE, late gadolinium enhancement; MT-FIDDLE, magnetization-transfer flow-independent dark-blood delayed enhancement

In this prospective comparative study, 110 STEMI patients enrolled in the MAgnetic Resonance IN Acute STEMI (MARINA-STEMI) trial (NCT04113356) between May and December 2021 (Fig. 1). See inclusion and exclusion criteria in the “Methods” section of the Supplementary material.

To rule out a possible influence of the time delay between CA administration and LGE imaging on the infarct volume, eleven additional STEMI patients were examined twice with the “bright-blood”-LGE sequence with different time delays between the acquisitions.

### CMR imaging

CMR exams were performed on a 1.5-T scanner (Magnetom AVANTO^fit^, Siemens) within the first week, 4 months and 12 months after STEMI. All acquisitions were performed under breath-hold conditions; details regarding CMR protocol and post-processing have been published previously [16].

“Bright-blood”-LGE images were acquired 10 min after intravenous administration of 0.15 mmol/kg of Gd-DO3A-butriol (Gadovist®, Bayer Vital) using an ECG-triggered, balanced steady-state free precession (bSSFP) based phase-sensitive inversion recovery (PSIR) sequence [17, 18] with short-axis slices covering the entire left ventricle (LV).

The prototype MT-FIDDLE sequence, as described by Kim et al [9] uses a bSSFP readout and was acquired immediately after the “bright-blood”-LGE sequence in the same short-axis slice positions. The protocol parameters of “bright-blood”-LGE and MT-FIDDLE sequence are detailed in the “Methods” section of the Supplementary Material.

### Image analysis

#### Subjective image quality

Subjective image quality parameters were rated by an observer with 5 years of CMR experience, blinded to clinical data as well independently of other CMR sequences, in particular to the other late enhancement sequence. Overall image quality, presence of artefacts as well as both, confidence in scar segmentation and in blood-pool bordering of “bright-blood”-LGE and MT-FIDDLE were visually assessed using a Likert scale, as in detail presented in the “Methods” section of the Supplementary material. For both LGE methods, the transmural scar extent was split into quartiles (1–24%, 25–49%, 50–74% and 75–100%) and the number of LGE affected LV segments was expressed in the standard American Heart Association 17-segment model [19]. Presence of papillary muscle enhancement, of microvascular obstruction (MVO) in the acute stage of STEMI as well as of involvement of the right ventricular (RV) wall was visually assessed. A randomly determined sample of 30 study participants (ten patients in each group with acute, subacute and chronic STEMI) was evaluated twice by the same and once by a second reader with 14 years of CMR experience to evaluate intra-observer and inter-observer variability.

#### Objective scar detectability

Objective scar detectability measures were derived from PSIR images of both LGE methods

Using Picture Archiving and Communication System (PACS) workstation (IMPAX EE workstation, Agfa Healthcare). Regions of interest were placed into the LV blood-pool, the RV blood-pool, the remote myocardium and the region of enhancement of the infarcted myocardium to derive mean signal intensities and standard deviations (SDs) of respective tissues. The detectability index (DI) [20] between scar and healthy myocardium or left ventricular blood was calculated using the following equation,

$${DI}=\,\frac{\left|{{SI}}_{1}-\,{{SI}}_{2}\right|}{\sqrt{{{SD}}_{1}^{2}+\,{{SD}}_{2}^{2}}}$$

where SI_1_ and SI_2_ are the mean signal intensities of the respective tissues and SD_1_ and SD_2_ are the associated standard deviations (SD) [21–23]. To ensure applicability of the DI, the ratio SI/SD was calculated for all regions of interest, which should have values > 3 to guarantee a probability distribution not far from Gaussian [24]. See Supplementary material.

#### Evaluation of infarct volume

Standard post-processing software (Circle Cardiovascular Imaging: cvi42) was used for infarct sizing analyses on both, short-axis “bright-blood”-LGE and MT-FIDDLE PSIR images. The infarct volume was calculated by FWHM method and SD method on both, “bright-blood”-LGE and MT-FIDDLE images. See the detailed description in the “Image analysis” section of the Supplementary Material.

### Statistical analysis

Statistical analysis was conducted using R Project for Statistical Computing (version 3.6.3, R Foundation for Statistical Computing). The statistical tests carried out are described in the “Methods” section of the Supplementary material.

## Results

### Patients and CMR characteristics

A total of 110 patients with a median age of 59 (IQR 54–66) years were scanned with “bright-blood”-LGE and MT-FIDDLE, CMR was performed on day 4 (IQR 3–5) after acute reperfused STEMI (33 patients (eight women)) as well as 4 months (133 days; (IQR 125–140)) (39 patients (seven women)) and 12 months (369 days; IQR 366–372) after STEMI (38 patients (six women)). Clinical characteristics of the patient population are summarized in Table 1.

**Table 1:** Clinical characteristics of the study population

Patient characteristics	(*n* = 110)
Age, years	59 (54–65)
Female, *n* (%)	21 (19.1)
Body mass index, kg/m²	25.2 (23.4–28.4)
Cardiovascular risk factors	
Hypertension, *n* (%)	43 (39.4)
Hyperlipidemia, *n* (%)	48 (44)
Pack years	20 (0–35)
Diabetes mellitus, *n* (%)	8 (7.3)
Family history of CAD, *n* (%)	22 (20.2)
Peak hs-cT, ng/L	4667 (2179.7–7214.5)
Peak CK, U/L	1861 (1136.7–3468.2)
Infarct-related artery	
LAD, *n* (%)	53 (43.8)
LCX, *n* (%)	15 (12.4)
RCA, *n* (%)	42 (34.7)
LV-EF (%)	47.1 (39.8–54.5)
IS, g (FWHM)	14.5 (7.7–21.1)
Infarct Transmurality %	
1–25, *n* (%)	1 (0.9)
26–50, *n* (%)	7 (6.4)
51–75, *n* (%)	25 (22.7)
76–100, *n* (%)	77 (70)
MVO, g	2.8 (1–5)

LV ejection fraction was determined from cine short-axis images as described in reference [17]. Infarct Transmurality refers to the evaluation with “bright-blood” LGE

*n* number, *CAD* coronary artery disease, *Hs-cT* high-sensitivity cardiac troponin T (during hospitalization for acute ST-elevation myocardial infarction), *CK* creatine kinase (during hospitalization for acute ST-elevation myocardial infarction), *LAD* left anterior descending artery, *LCX* left circumflex artery, *RCA* right coronary artery, *IS* infarct size, *LVEF* left ventricular ejection fraction, *MVO* microvascular obstruction

Time from contrast agent administration to acquisition was 10.7 ± 2.0 min for the “bright-blood”-LGE and 20.0 ± 3.1 min for the MT-FIDDLE sequence. The median time between the acquisition of the “bright-blood”-LGE and MT-FIDDLE was 9.0 min (IQR 7.7–10.4 min). The acquisition of the “bright-blood”-LGE took 3.1 ± 1.1 min and 4.9 ± 0.8 min for FIDDLE (*p* < 0.001). The inversion time used for “bright-blood”-LGE was 263 ± 22 ms and 193 ± 29 ms for MT-FIDDLE.

### Subjective image quality

**Fig. 2:**
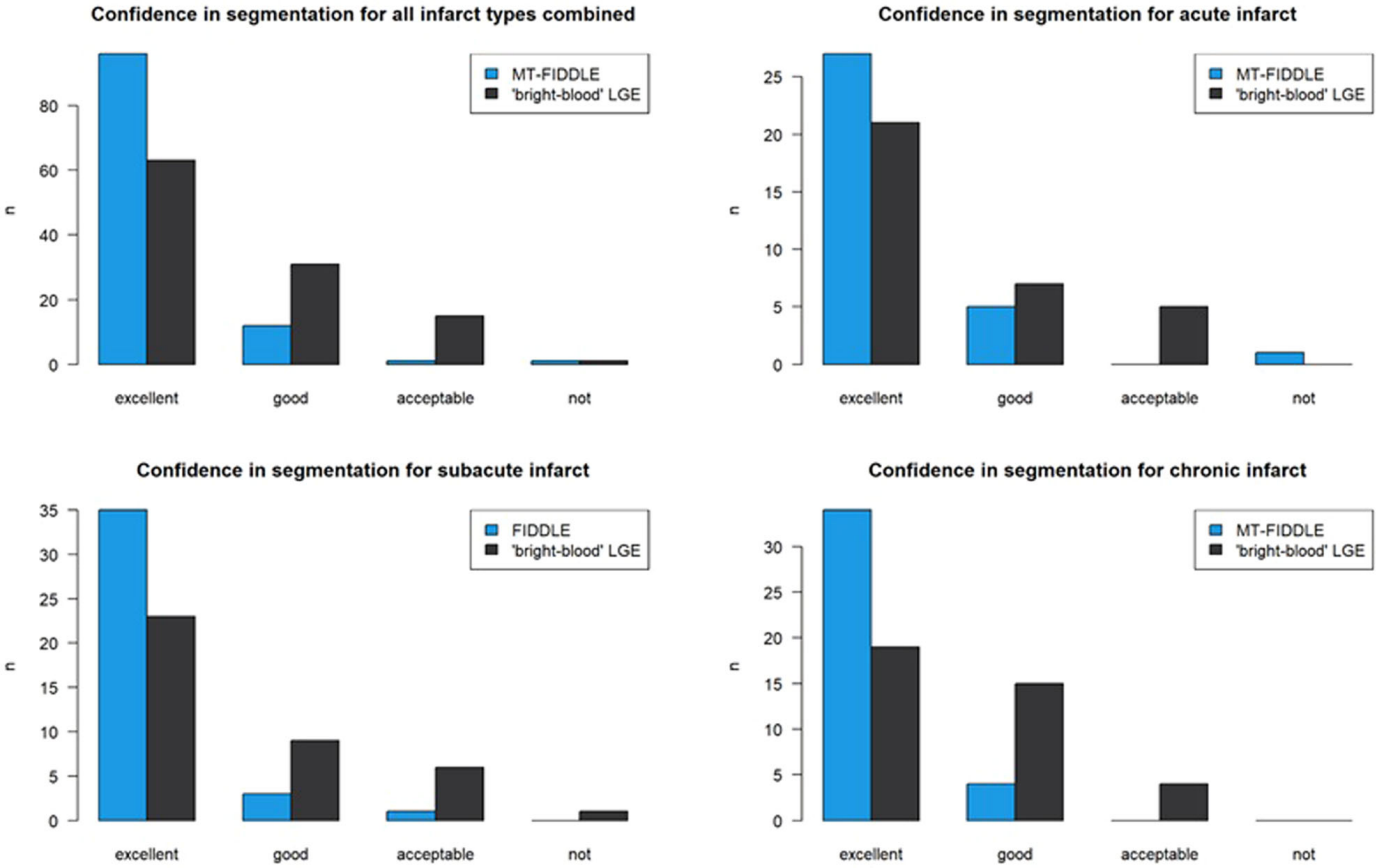
Confidence in delineation of infarct from blood for all infarct types combined between “bright-blood”-LGE and MT-FIDDLE. The number of patients with excellent confidence in infarct segmentation clearly increased overall, as well as for the individual infarct types. The difference in confidence in infarct segmentation between “bright-blood”-LGE and MT-FIDDLE was highly significant overall (*p* < 0.001), for subacute (*p* = 0.00167) and chronic (*p* < 0.001) infarcts, but not yet significant for acute infarcts (*p* = 0.083)

The overall image quality of both “bright-blood”-LGE and MT-FIDDLE was rated as excellent without statistically significant difference between sequences (*p* = 0.997). No images were assigned as non-diagnostic. Excellent image quality without artifacts was noted in 89 patients for MT-FIDDLE and in 83 patients for “bright-blood”-LGE. Compared to “bright-blood”-LGE, for the MT-FIDDLE sequence, the number of patients with excellent confidence in scar segmentation clearly increased overall (96 vs. 57 patients), as well as for the different stages of myocardial infarction (see Fig. 2). In addition, confidence in scar segmentation was significantly higher for MT-FIDDLE compared to “bright-blood”-LGE overall (*p* < 0.001), for subacute (*p* = 0.002) and chronic (*p* < 0.001) infarcts, but not significantly for acute infarcts (*p* = 0.083).

**Fig. 3:**
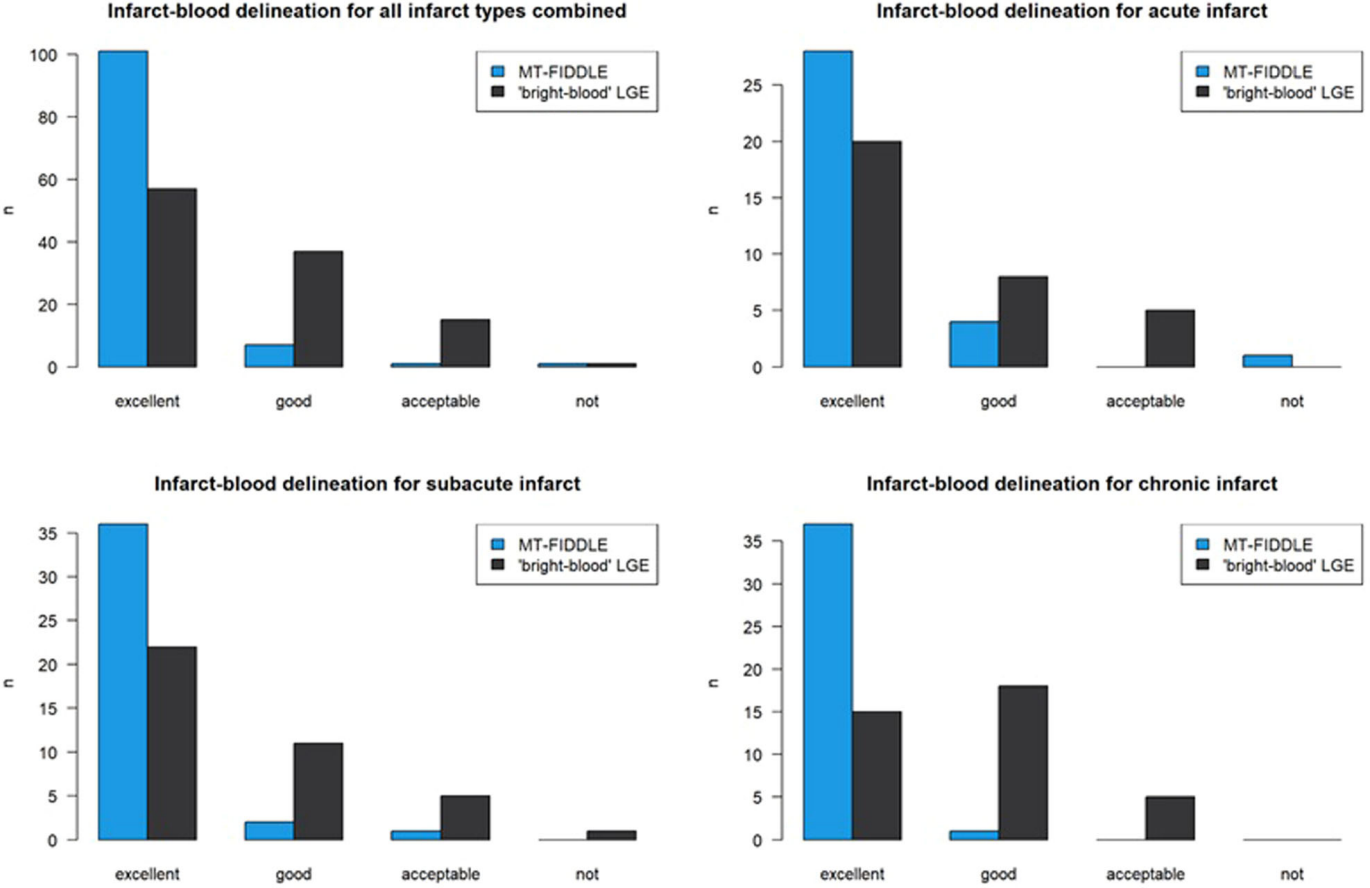
Confidence in delineation of infarct from blood for all infarct types combined between “bright-blood”-LGE and MT-FIDDLE. The number of patients with excellent confidence in infarct-blood delineation clearly increased over all, as well as for the individual infarct types. The difference in confidence in infarct-blood delineation between “bright-blood”-LGE and MT-FIDDLE was highly significant overall (*p* < 0.001), significant for acute infarcts (*p* = 0.026) and highly significant for subacute (*p* < 0.001) and chronic (*p* < 0.001) infarcts

The number of patients with excellent confidence in blood-pool bordering was also clearly higher for MT-FIDDLE (101 vs. 63 patients; see Fig. 3) and the difference was highly significant overall (*p* < 0.001), significant for acute infarcts (*p* = 0.026) and again highly significant for subacute (*p* < 0.001) and chronic infarcts (*p* < 0.001).

The visual scar transmurality for both sequences was 76–100% in 77 patients (70.0%), 51–75% in 25 patients (22.7%) and 26–50% in 7 patients (6.4%). One patient with an infarction affecting less than 26% transmurality was shown by MT-FIDDLE, while “bright-blood”-LGE missed this single infarction (0.9%). Although not significant, MT-FIDDLE revealed more LGE affected AHA segments in *n* = 16 (14.6%) patients (*p* = 0.633). Papillary muscle enhancement was significantly (*p* < 0.001) more often detected in MT-FIDDLE sequence as compared to “bright-blood”-LGE; see details on affected papillary muscles in the “Results” section of the Supplementary material.

MVO was detected in a total of 15 patients with acute STEMI in both, MT-FIDDLE and “bright-blood”-LGE. RV LGE involvement was visible in 7 patients, both in MT-FIDDLE as well as in “bright-blood”-LGE. There were no intraventricular thrombi detected in both sequences.

Inter- and intra-observer agreement for overall image quality, confidence in scar segmentation, confidence in blood-pool bordering and scar transmurality was excellent for all measurements in both sequences (overall agreement: 90–100%, Gwet’s AC1: 0.95–1.0).

### Objective scar detectability

To assess the homogeneity of blood-pool suppression in MT-FIDDLE compared to “bright-blood”-LGE, the mean signal intensity between LV and RV blood pools was compared and showed no significant difference (*p* = 0.953).

For LGE the mean ratio of Si/SD was 49.1 (range: 11.4–985.6) for scar, 46.4 (range: 22.0–96.1) for normal myocardium and 83.4 (range: 38.4–189.7) for left ventricular blood. For MT-FIDDLE it was 133.1 (range: 50.6–727.7), 221.6 (range: 57.9–553.5) and 388.6 (range: 182.0–1081.1), respectively, justifying the assumption of a probability distribution not far from Gaussian [20, 24] needed for the applicability of DI.

The DI between scar and blood was significantly higher for MT-FIDDLE (8.9 ± 5.5) as compared to “bright-blood”-LGE (2.0 ± 1.5; *p* < 0.001). On the other hand, the DI between scar and remote myocardium was higher for “bright-blood”-LGE (7.4 ± 3.2) as compared to MT-FIDDLE (5.2 ± 2.0; *p* < 0.001) and the DI between blood and healthy myocardium was also higher for “bright-blood”-LGE (10.1 ± 3.2) compared to MT-FIDDLE (4.5 ± 2.4; *p* < 0.001). The differences were significant even after adjustment for the possible influence of time delay using an ANCOVA test (for details, see Supplementary material).

### Infarct and MVO volumes

For the FWHM technique there was a gradual decrease in the concordance of infarct sizes between MT-FIDDLE and “bright-blood”-LGE from the group with excellent confidence in infarct segmentation on “bright-blood”LGE (CCC = 0.96 (95% CI: 0.94–0.98)) to those with good (CCC = 0.96 (95% CI: 0.91–0.98)) and acceptable confidence (CCC = 0.93 (95% CI: 0.88–0.96)), while the difference was significant (*p* < 0.001) only between excellent and acceptable and good and acceptable confidence in “bright-blood”-LGE infarct segmentation. The concordance over all infarcts without differentiation into different confidence classes was still very high (CCC = 0.96 (95% CI: 0.94–0.97), bias: 0.0 mL (*p* = 0.980)).

**Fig. 4:**
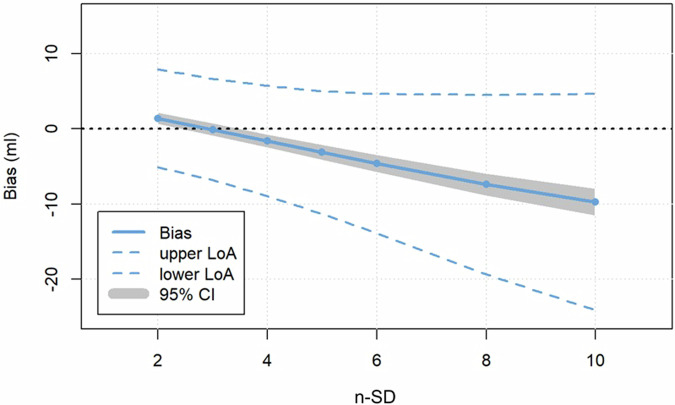
Bias (mean difference) between infarct volumes obtained from “bright-blood”-LGE and MT-FIDDLE sequences by Bland-Altmann analysis for different SD-values used for scar segmentation based on the standard deviation method. Only patients were included where the confidence in scar segmentation for “bright-blood”-LGE was excellent. For 3-SD, the lowest bias between “bright-blood”-LGE and MT-FIDDLE sequence was found

For the SD-thresholding techniques, the best concordance of infarct volumes was found for the subgroup with excellent confidence in infarct segmentation on “bright-blood”-LGE between the 5-SD technique on “bright-blood”-LGE and the 3-SD technique on MT-FIDDLE (CCC = 0.96, (95% CI: 0.93–0.97); bias −0.11 mL; *p* = 0.808), see Fig. 4. However, also for the overall patient cohort infarct volume as assessed by the 5-SD technique on “bright-blood”-LGE agrees best with the 3-SD-technique on MT-FIDDLE (CCC = 0.94, (95% CI: 0.91–0.96); bias 0.21 mL; *p* = 0.586). Excellent intra- and interrater agreement was found regarding scar volume between both LGE sequences using the FWHM method (overall agreement intra-class correlation coefficient = 0.89–0.99).

By using the FWHM method, the manually included MVO volume was significantly lower in the MT-FIDDLE sequence as compared to “bright-blood”-LGE sequence (*p* = 0.025, CCC = 0.77 (0.48–0.91)).

### Comparison of infarct volume between acute, subacute and chronic STEMI by FWHM and 5-SD method

Although there was no difference in scar volume between MT-FIDDLE and “bright-blood”-LGE for all infarct groups combined, we obtained slightly higher scar volumes with “bright-blood”-LGE for the subgroup of acute infarcts. The median difference for acute infarcts was 0.7 mL (range: −5.7 to 16.0 mL) for FWHM and 4.0 mL (range: −2.4 to 24.5 mL) for the 5-SD method, respectively, and was significantly different from zero in both cases (*p* = 0.02). In the subacute group the difference between “bright-blood”-LGE and MT-FIDDLE was significantly lower as compared to the acute group (*p* < 0.001 for FWHM and *p* = 0.001 for 5-SD) with a median difference of −1.2 mL (*p* < 0.01; range: −9.0 to 4.7 mL) for FWHM and 1.9 mL (*p* = 0.001; range: −8.0 to 10.6 mL) for the 5-SD method. Also in the chronic group, a significantly lower difference between “bright-blood”-LGE and MT-FIDDLE was observed (*p* = 0.03 for FWHM and *p* < 0.001 for 5-SD) with a median difference of 0.09 mL (*p* = 0.6; range: −6.7 to 7.8 mL) for FWHM and 0.5 mL (*p* = 0.02; range: −5.8 to 11.8 mL) for the 5-SD method. Between subacute and chronic group, the difference between “bright-blood”-LGE and MT-FIDDLE was not statistically significant (*p* = 0.439 for FWHM and *p* = 0.917 for 5-SD).

### Impact of “time delay” between sequences on DI and infarct volume

A detailed evaluation of the influence of time delay between the sequences on DI and infarct volume can be found in the Supplementary material. In short, eleven patients with STEMI were examined twice with the “bright-blood”-LGE sequence with different time delays between the acquisitions (median delay 7.0 min (IQR: 5.8–8.4 min)). No statistically significant difference between both acquisitions was found for DI between scar and normal myocardium (*p* = 0.814) as well as between scar and left ventricular blood (*p* = 0.305), and no significant correlation between DI and the time after CA injection was observed. Finally, no significant difference in scar volumes between both measurements (*p* = 0.434) and no correlation between the difference in infarct volume of both measurements and the time delay was observed (*r* = 0.47, *p* = 0.140) (see Supplementary Fig. 2).

**Fig. 5:**
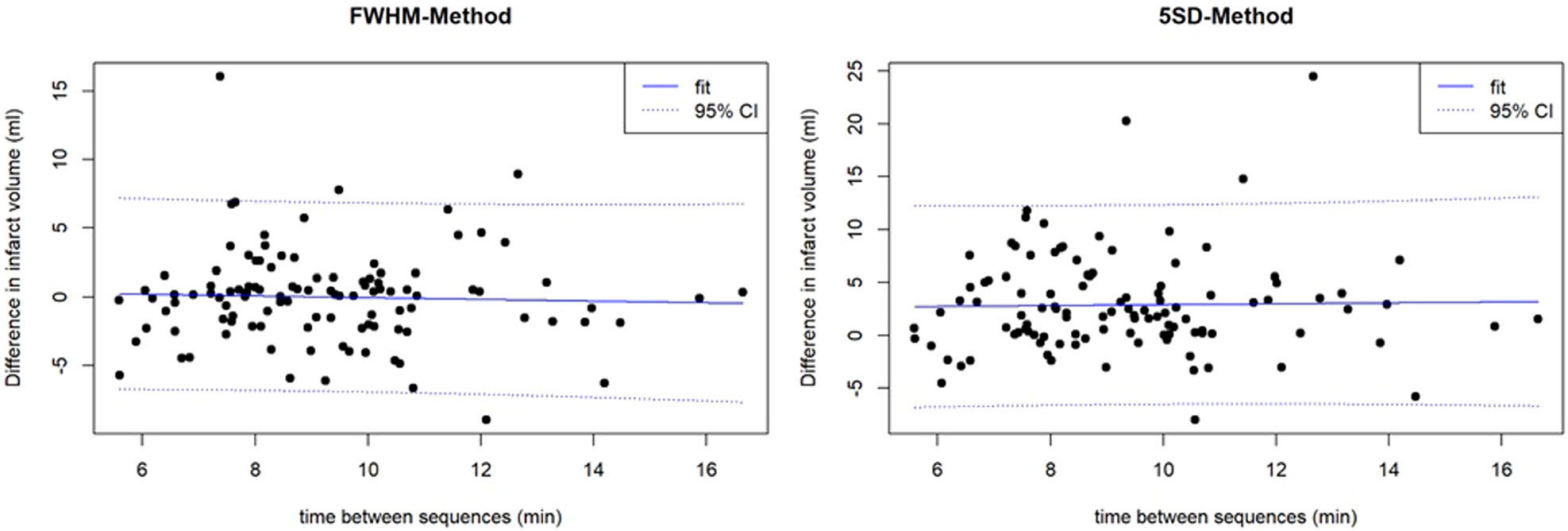
Difference in scar volume between “bright-blood”-LGE and MT-FIDDLE sequence as a function of the time delay between the sequences. Due to clinical workflow reasons, the “bright-blood”-LGE images were always acquired prior to the MT-FIDDLE images. No significant correlation between volume difference and time delay was observed both for the FWHM method (*r* = 0.04, *p* = 0.679) and the 5 SD method (*r* = 0.02, *p* = 0.8377)

Finally, for our patients with “bright-blood”-LGE followed by MT-FIDDLE no statistically significant correlation between the difference of infarct volume (“bright-blood”-LGE vs. MT-FIDDLE) and the time delay was observed both for the FWHM method (*r* = 0.04, *p* = 0.679) and the 5 SD method (*r* = 0.02, *p* = 0.838); see Fig. 5. Details regarding the correlation between DI and the delay between “bright-blood”-LGE and MT-FIDDLE can also be found in the Supplementary material.

## Discussion

The main findings of the study can be summarized as follows:

1. MT-FIDDLE showed significantly higher confidence scores in scar segmentation and in blood-pool bordering compared to the “bright-blood”-LGE sequence.
2. MT-FIDDLE provides significantly higher DI of ischemic myocardium versus blood-pool and significantly lower DI of ischemic versus remote myocardium in comparison to “bright-blood”-LGE.
3. For infarct volume quantification in MT-FIDDLE, the 3-SD technique for thresholding results in best agreement with the established 5-SD technique on “bright-blood”-LGE. The FWHM technique provides comparable infarct volumes in MT-FIDDLE and “bright-blood”-LGE.
4. MT-FIDDLE more often revealed papillary muscle LGE.

**Fig. 6:**
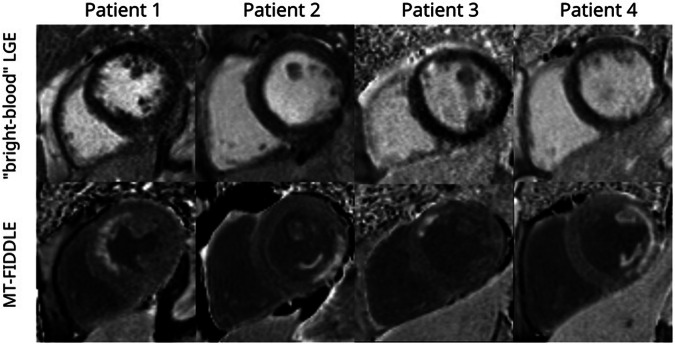
For patient 1, hyperenhancement by MT-FIDDLE clearly identified infarcted myocardium in the anterior wall and for patient 2 in the posterior wall with extension to posterolateral papillary muscle. MT-FIDDLE detected a small infarct in the anterior wall of patient 3 and a lateral wall infarct with extension to the posterolateral and the anterolateral papillary muscle in patient 4. In none of these four patient cases did “bright-blood” hyperenhancement clearly identify all infarcted regions. Infarct identification by “bright-blood”-LGE was particularly poor at the subendocardial border

The “bright-blood”-LGE is currently the gold standard imaging technique for diagnosis of MI. To overcome the major limitation of reduced contrast between blood-pool and myocardial scar in the “bright-blood”-LGE imaging several “dark-blood”-LGE methods were introduced [9–14]. The novel dark-blood sequence MT-FIDDLE with MT-preparation recently reported and histologically validated by Kim et al demonstrated diagnostic superiority over the “bright-blood”-LGE sequence with higher sensitivity and accuracy as well as better agreement for infarct volume measurements [9] (see Fig. 6).

To test the clinical performance of the MT-FIDDLE sequence, we conducted a prospective study on a large population of STEMI patients (*n* = 110). In our study, MT-FIDDLE revealed significantly higher confidence scores in scar segmentation and in blood-pool bordering in comparison to the “bright-blood”-LGE sequence along with excellent inter- and intra-observer agreement. This was also supported by the obtained DI values. According to the Rose criterion, the object detection threshold of DI is between 3 and 5, depending on the observer’s expertise [20, 25]. For “bright-blood”-LGE, DI between scar and blood pool was clearly below this threshold (DI: 2.0 ± 1.5) while it had a value above the threshold for MT-FIDDLE (DI: 8.9 ± 5.5). This difference of DI between scar and blood-pool was shown to be highly significant, explaining the higher reader confidence for MT-FIDDLE as compared to “bright-blood”-LGE. In contrast, the DI between ischemic and remote myocardium was significantly lower for MT-FIDDLE compared to the “bright-blood”-LGE sequence, mostly due to the dark-gray appearance of the remote myocardium in the MT-FIDDLE sequence.

Furthermore, MT-FIDDLE resulted in the detection of both, more LGE (*n* = 16) affected AHA segments and more frequent papillary muscle enhancement compared to the “bright-blood”-LGE sequence. Thereby, our results regarding papillary muscle enhancement are in line with the recently published study by Wendell et al who found MT-FIDDLE to be more sensitive (100% vs 57%, *p* < 0.001) and accurate (100% vs 80%, *p* = 0.01) than “bright-blood”-LGE in identifying papillary muscle infarction [26].

Foley et al suggested routine adoption of a “blood-nulled” PSIR approach to be appropriate for LGE imaging because of its significantly higher scar-to-blood CNR compared to routine myocardial nulling and its significantly higher reader confidence scores compared to routine myocardial nulling and T1-ρ-LGE [15]. Unlike the T1-ρ-LGE method, the MT-FIDDLE has a black-blood and dark-gray appearance of myocardium on images resulting in significantly higher scar-to-blood contrast, thus facilitating the detection of subendocardial scars. Despite the dark-gray appearance of the myocardium and reduced DI values between ischemic and remote myocardium on MT-FIDDLE images, we found comparable infarct volumes as assessed on MT-FIDDLE and “bright-blood”-LGE images measured by the FWHM technique. However, the reduced DI values between ischemic and remote myocardium on MT-FIDDLE might render the usage of MT-FIDDLE for non-ischemic LGE imaging disadvantageous.

Most novel “dark-blood” CMR methods have been compared to “bright-blood”-LGE, as was also the case in the recent study by Foley et al [15]. However, a histopathology validated comparison was only performed for FIDDLE [9] and “blood-nulled”-PSIR-LGE [27], both studies used manual contouring as myocardial scar quantification method. In addition, a recently published study reported the results of semi-automated myocardial scar quantification techniques including signal threshold versus reference mean and FWHM as well as manual contouring for “blood-nulled”-PSIR-LGE compared with histopathology [28]. However, no studies investigating the effect on semi-automatic scar quantification methods were tested on MT-FIDDLE images. Therefore, we investigated the most suitable semi-automatic scar quantification method for MT-FIDDLE sequence in comparison to “bright-blood”-LGE in clinical practice context.

For the SD-based method of infarct quantification, we found the threshold of 3-SD for MT-FIDDLE to result in the best agreement to the well-established 5-SD method used for “bright-blood”-LGE sequences. Thereby, the reduced number of SDs is most probably explained by the observed lower DI between scar and myocardium for MT-FIDDLE. In contrast, for the MVO volume, it was shown that the application of the 5-SD technique for both sequences, MT-FIDDLE and “bright-blood”-LGE, yielded largely comparable results, which most likely is explainable by the influence of the delay between contrast injection and time of scanning on the MVO volume. However, the FWHM method was not as suitable as 5-SD method to evaluate the MVO volume. Since the MT-FIDDLE variant used in this study uses MT-preparation, which is not dependent on blood flow, we did not detect any “slow-blood-flow” artefacts within the blood-pool of the LV or RV on short-axis images, which is in accordance with Kim et al [9]. Recently, Jenista et al, compared two different preparation schemes for FIDDLE, T2-preparation and MT-preparation, in 35 patients. This study showed that FIDDLE with MT-preparation results in fewer bright blood-pool artefacts, e.g., signs of incomplete blood-pool suppression and thus in a more homogenous blood-pool suppression [29], which fits to our observation of non-significant signal difference between the LV and RV blood-pool.

The infarct volume between subacute and chronic STEMI did not differ when compared between the “bright-blood”-LGE and MT-FIDDLE. However, the infarct volume of acute STEMI was significantly higher in “bright-blood”-LGE compared to MT-FIDDLE. The volume mismatch between “bright-blood”-LGE and MT-FIDDLE in acute STEMI might be due to the MT-preparation of the MT-FIDDLE sequence. For edema tissue, the MT-preparation should lead to slightly larger magnetization compared to non-edematous tissue and thus after the inversion pulse to slightly less signal in the PSIR images, which possibly could explain the observed lower volume in acute STEMI [30–33].

Our study had some limitations: In our clinical setting, MT-FIDDLE images were obtained around 20 min after initial contrast agent injection, with a time delay between “bright-blood”-LGE and FIDDLE of around 9 min. While the measurement time of MT-FIDDLE was well within the range in which LGE sequences could be performed [26], the time delay between “bright-blood”-LGE and MT-FIDDLE might have affected their comparison. However, as there was no significant correlation of DIs or infarct volumes with time delay for “bright-blood”-LGE measurements (in accordance with Doltra et al [34]), the impact of the time delay between “bright-blood”-LGE and MT-FIDDLE might, however, be considered as small. This conclusion is further supported by the result that significant differences in DIs of both sequences remain after adjustment for time delay and that differences in infarct volumes do not depend significantly on the time delay between “bright-blood”-LGE and MT-FIDDLE.

In addition, also no dependence of infarct volume on time delay was found for “bright-blood”-LGE in eleven additional patients. Although the significance of this finding is limited due to the low number of additional patients, it is nevertheless in line with the paper of Wagner et al [35], who also found no effect of time delay if inversion time (TI) of the LGE sequence was adjusted individually, as was the case in our study. However, additional studies with MT-FIDDLE measured twice and with “bright-blood”-LGE and MT-FIDDLE in opposite order might have allowed a more thorough analysis on the behavior of MT-FIDDLE properties with respect to the time between contrast agent administration and its acquisition.

Another important limitation of the work is that the imaging parameters of the two LGE sequences were not fully matched. Although the acquisition windows during one heartbeat of both protocols were matched to ensure similar blurring effects due to heart motion, the choices of the MT-FIDDLE protocol, according to Kim et al [9], and of the “bright-blood”-LGE protocol as the standard protocol of the MARINA-STEMI project may have resulted in a slight disadvantage for the “bright-blood”-LGE in quantitative image quality measures.

According to the study design, the observer was blinded to clinical details but biased in that all study participants had STEMI, and no non-infarcted subjects were part of the study cohort. In our study, chronic infarct scars were defined as 12 months after the acute event. We did not include patients with chronic infarct scars older than 12 months. The same patient could not be examined longitudinally at three time points (during the first week after acute STEMI and 4 and 12 months thereafter) due to the limited time in which the MT-FIDDLE prototype sequence was available to us. Moreover, the vast majority of STEMI patients in this study showed > 75% visual scar transmurality, therefore our findings are not generalizable for smaller, subendocardial infarcts. However, in our opinion, also these groups of patients with chronic ischemic cardiomyopathy, as well as patients with discrete subendocardial infarcts, would specifically benefit from additional imaging with MT-FIDDLE sequence, as the differentiation of blood-scar-border in “bright-blood”-LGE may be highly reduced due to the thinned myocardium.

Evaluation and measurements of “bright-blood”-LGE and MT-FIDDLE sequences were done on PSIR-reconstructed images, as it is less sensitive to non-optimal TI-selection and achieves a consistent contrast compared to magnitude-reconstructed images, which are highly sensitive to selected TI-time [17].

The clinical practicability and efficiency of the MT-FIDDLE sequence are essentially equivalent to “bright-blood”-LGE sequence. The spatial resolution and the acquisition window during one heartbeat of both protocols were matched. The slightly longer scan time of the MT-FIDDLE sequence compared to the “bright-blood”-LGE sequence reflects the slightly longer scan time per slice of the MT-FIDDLE sequence (8–10 s vs. 5–6 s), which was chosen to equal the MT-FIDDLE protocol in Kim et al [9] and, as previously stated, may have resulted in a slight disadvantage for “bright-blood”-LGE in quantitative image quality assessment. Finally, for MT-FIDDLE, the same amount of contrast agent can be used, and there is no need for additional post-processing. Therefore, this sequence can be successfully implemented in clinical routine as an additional sequence for further investigation of the ischemic scar extent in case of inconclusivity of “bright-blood”-LGE, especially at the subendocardial border. However, at the beginning of implementing the MT-FIDDLE, due to sequence novelty, the interpretation of the MT-FIDDLE scout can lead to minimal scan time delay.

Whereas the blood-nulled PSIR-LGE method is a freely and widely available dark-blood-LGE approach, at the moment, however, the dark-blood sequence with additional magnetization preparation schemes is not yet available commercially, thus limiting their widespread in clinical practise. Nevertheless, MT-FIDDLE indeed remains an excellent approach (if available) as it also demonstrates a superior visualization and quantification of ischemic scar compared to bright-blood-LGE.”

## Conclusion

The limited conspicuity at the subendocardial border in the “bright-blood”-LGE sequence may lead to myocardial infarct scars being missed or misjudged. The novel dark-blood sequence FIDDLE with MT-preparation facilitates the assessment of myocardial infarct scars at subendocardial border and provides greater diagnostic confidence compared to “bright-blood”-LGE sequence.

## Supplementary information

Supplementary Material

## Abbreviations

CCC: Concordance correlation coefficient
Cl: Confidence interval
CMR: Cardiac magnetic resonance
DI: Detectability index
FWHM: Full-width at half-maximum
IQR: Interquartile range
LGE: Late gadolinium enhancement
LV: Left ventricle
MT: Magnetization transfer
MT-FIDDLE: Magnetization-transfer flow-independent dark-blood delayed enhancement
MVO: Microvascular obstruction
PSIR: Phase-sensitive inversion recovery
RV: Right ventricle
SD: Standard deviation
STEMI: ST-elevation myocardial infarction
TI: Inversion time
