# Supplementary material for: Magnetization-transfer flow-independent dark-blood delayed enhancement cardiac MRI optimizes discrimination of ST-elevation myocardial infarct borders
Source: Eur Radiol. 2024 Dec 5;35(6):3030–41. doi: 10.1007/s00330-024-11192-7 (PMC12081512; doi:10.1007/s00330-024-11192-7)
Supplement: Supplementary file 1 — Supplementary Material [file 330_2024_11192_MOESM1_ESM.pdf]

Supplementary Material

The following supplemental sections are provided as additional information:

Supplementary METHODS:

Study population

Inclusion criteria were first time STEMI according to the redefined European Society of Cardiology/American College of Cardiology committee criteria [1], revascularization by primary percutaneous coronary intervention (pPCI) within 24 hours after the onset of symptoms, an estimated glomerular filtration rate > 30 mL/min per 1.73 m2, and Killip class < 3 at time of CMR. Exclusion criteria were age < 18 years, any history of previous myocardial infarction or coronary intervention, and any contraindication to CMR examination.

CMR Imaging

Protocol parameters of the “bright-blood” late gadolinium enhancement (LGE) sequence were: slice thickness: 8 mm; interslice gap: 1.5 mm; field of view (FOV): 360 mm × 349 mm, (Percent phase FOV: 96.9%); acquisition matrix: 256 x 184 (phase resolution: 72%; no phase partial Fourier; phase oversampling: 6%); voxel size: 1.4 mm × 1.9 mm × 8.0 mm; repetition time (TR): 2.9 ms; echo time (TE): 1.2 ms; inversion time (TI): 140-340 ms; flip angle: 45°; number of segments: 65; receive bandwidth: 780 Hz/Px. Using a “Look-Locker” scan (TI scout) TI was adjusted individually for every patient to null the signal of remote myocardium.

The prototype MT-FIDDLE sequence, as described by Kim et al. [2] was acquired immediately after the “bright-blood” LGE sequence in the same short-axis slice positions. Protocol parameters were slice thickness: 8 mm; interslice gap: 1.5 mm; FOV: 360 mm x 349 mm (Percent phase FOV: 96.9%); acquisition matrix: 256 x 189 (phase resolution: 74%; phase

partial Fourier: 7/8; phase oversampling: 66%); voxel size: 1.4 mm x 1.9 mm x 8.0 mm; TR: 3.3 ms; TE: 1.4 ms; TI: 150-440 ms, maximized while remote myocardium magnetization exceeds blood pool magnetization; flip angle: 55°; number of segments: 55; receive bandwidth: 515 Hz/Px. Due to the slightly longer TR the number of segments were adjusted so that the acquisition window during one heart beat was similar for both sequences to ensure similar blurring effects due to heart motion. The MT-FIDDLE sequence includes a leading magnetization preparation module which differentially saturates tissue compared to blood, followed by a spatially non-selective inversion recovery pulse. For differential tissue saturation a series of 19 off-resonance (600Hz) MT pulses (duration: 6.4ms; flip angle: 500°) with an inter-pulse delay of 3ms are applied. The MT pulse suppresses the magnetization of myocardium (normal and infarcted) to a greater extent than that of blood, so that after the inversion pulse the magnetization of myocardium is less negative than that of blood. At a properly selected inversion time a situation can be achieved where the magnetization of blood is more negative than normal myocardium. The sequence performs an inline phase sensitive PSIR reconstruction procedure which produces magnitude and PSIR images. On the PSIR image blood will then appear darker than myocardium Thereby only the PSIR image representing the “dark-blood” MT-FIDDLE image and the magnitude image was ignored for the subsequent evaluation. To adjust TI for the MT-FIDDLE sequence a special TI scout sequence was used with its parameters set to exactly reproduce the magnetic saturation and recovery behaviour of the final sequence, however, producing a series of images with varying TI. The optimal TI value was selected from the image with the longest TI that still shows lower signal in blood as compared to myocardium.

Image analysis

Subjective image quality

Artefacts on both sequences were evaluated by using a 5-point Likert scale (0 = no artefacts, 1 = weak artefacts, 2 = moderate artefacts, 3 = strong artefacts, 4 = non diagnostic). Both,

confidence in scar segmentation as well as in blood pool bordering were rated by using a 4-point Likert scale (1 = excellent delineation, 2 = good delineation, 3 = acceptable delineation, 4 = not to delineate).

Evaluation of Infarct volume

For the FWHM method the scar threshold was chosen in the middle (50%) between the maximal signal within the scar and the minimum of remote myocardium. For the SD method, a region of interest (ROI) was drawn within the remote myocardium to define the mean reference signal intensity and its SD. For “bright-blood”-LGE images the scar threshold was chosen 5 SD above the mean reference signal intensity, as recommended by the Society for Cardiovascular Magnetic Resonance (SCMR) [3]. For the MT-FIDDLE images the threshold was varied between 2 and 10 SDs above the mean reference signal in order to evaluate the optimal threshold to quantify the infarct volume in agreement with the established 5-SD-approach for the “bright-blood”-LGE sequence. As mentioned above, in a sample of 30 randomly selected patients, intra-observer and inter-observer variability was also assessed regarding the evaluation of infarct volume.

For acute STEMI the volume of any MVO was recorded separately by manual planimetry using the FWHM method and the SD method on both, "bright-blood"-LGE and MT-FIDDLE images. In case of an MVO its volume was added to the hyperenhancing infarct mass.

Statistical analyses

Categorical variables are presented as frequencies and corresponding percentages. The Shapiro-Wilk test was applied to test for normal distribution. All results are expressed as mean ± SD (if normally distributed) or as median with interquartile range (IQR) (if not normally distributed).

Differences in quality or confidence scores were tested using the Kruskal-Wallis rank sum test. The concordance correlation coefficient (CCC) was used to evaluate the agreement between

measurements of two continuous variables. Possible biases were tested for significance using a one sample Student’s t-test. Differences between continuous variables were tested with paired Student's t-test or Wilcoxon-rank test, as appropriate. Correlations between variables were investigated by fitting a linear model. To compare linear models and to control for potential confounding variables (e.g. time delay between CA administration and image acquisition) Analysis of Covariance (ANCOVA) was used.

In order to avoid paradoxical results due to the presence of high agreement, intra- and inter-rater agreement for categorical variables were calculated using Gwet’s AC1 coefficient [4].

For all tests, a p value <0.05 was considered statistically significant.

Supplementary RESULTS:

Influence of time delay between contrast injection and image acquisition:

To investigate the influence of the delay between contrast application and acquisition of the “bright-blood”-LGE sequence on DI and the infarct volume, 11 patients with STEMI were examined twice with the “bright-blood”-LGE sequence. The median time between CA injection and first acquisition was 9.85min (IQR: 9.55 – 10.8min) and for the second acquisition 17.17min (IQR: 15.72 – 18.53min) leading to a median time delay between the acquisitions of 6.95 min (IQR : 5.8-8.4 min). As shown in figure 1suppl, no statistically significant difference was found between the first and second acquisition for either DI between scar and normal myocardium (p=0.814) or between scar and left ventricular blood (p=0.305) and also no significant correlation between DI and the time after CA injection was observed (DI between scar and normal myocardium: r=0.048/ p=0.833; DI between scar and left ventricular blood: r=0.163/ p=0.632). Finally, figure 3suppl shows, that no significant difference in scar volumes between both measurements (p=0.434) and no correlation between the difference of infarct volume (FWHM method) of both measurements and the time delay was observed (r=0.47, p=0.140). (see supplementary fig 2)

Analysing the data of all patients with „bright blood“ LGE followed by MT-FIDDLE acquisition no correlation of DI and time delay was found for „bright blood“ LGE between scar and normal myocardium (r=0.116, p=0.227), scar and blood (r=0.12, p=0.22) as well as normal myocardium and blood (r=0.027, p=0.776). For MT-FIDDLE a weak correlation was found between scar and normal myocardium (r=0.219, p=0.02), and normal myocardium and blood (r=0.2, p=0.03), but not between scar and blood (r=0.116, p=0.227). Comparing the linear models between „bright blood“ LGE and MT-FIDDLE using an ANCOVA test no significant difference in the slope of the regression lines was found (scar vs. normal myocardium: p=0.956; scar vs. LV blood: p=0.811; normal myocardium vs. LV blood: p=0.733), For all cases, ANCOVA showed a significant effect between „bright blood“ LGE and MT-FIDDLE ($p < 0.001$).

Subjective image quality

Papillary muscle enhancement was significantly ($p<0.001$) more often detected in MT-FIDDLE sequence (LGE of both papillary muscles in 27 patients, LGE of the anteromedial papillary muscle in 6 patients, LGE of the posterolateral muscle in 20 patients and no papillary muscle LGE in 57 patients) as compared to “bright-blood” LGE (LGE of both papillary muscles in 5 patients, LGE of the anteromedial papillary muscle in 6 patients, LGE of the posterolateral muscle in 20 patients and no papillary muscle LGE in 79 patients).

Supplementary DISCUSSION:

Several dark blood sequences, which are not dependent on blood movement, have been previously described showing results of improved conspicuity at the sub-endocardial border and blood pool. Liu et al. proposed a T2Prep-IR preparation method that uses combined T1-

and T2-weighting to produce images with improved contrast between blood-pool and sub-endocardial border of myocardial infarction [5]. Peel et al. investigated a method using a dual inversion-recovery pre-pulse, which improved scar to blood CNR and showed higher confidence scores compared with the conventional LGE sequence [6]. However, the blood-pool using these methods appeared “grey” on images and not “dark” as in FIDDLE. Moscogiuri et al., found a better LGE detectability in a small cohort of patients with known or suspected prior myocardial infarction compared to the conventional “bright blood” LGE method, when using “T1rho and magnetization transfer and INvErsion Recovery” (TRAMINER), a method that requires lower radiofrequency energy compared to MT-FIDDLE [7].

Kellman et al. used a combined inversion recovery and T2 preparation scheme with single shot steady state free precession imaging and respiratory motion correction to produce dark blood LGE images. The study showed a greatly improved delineation at sub-endocardial border and depiction of fibrosis in dark blood LGE imaging in 30 patients with sub-endocardial myocardial infarction as compared to bright blood LGE imaging [8].

REFERENCES

1 Ibanez B, James S, Agewall S et al (2017) 2017 ESC Guidelines for the management of acute myocardial infarction in patients presenting with ST-segment elevation. Rev Esp Cardiol (Engl Ed) 70:1082

2 Kim HW, Rehwald WG, Jenista ER et al (2018) Dark-Blood Delayed Enhancement Cardiac Magnetic Resonance of Myocardial Infarction. JACC Cardiovasc Imaging 11:1758-1769

3 Schulz-Menger J, Bluemke DA, Bremerich J et al (2020) Standardized image interpretation and post-processing in cardiovascular magnetic resonance - 2020 update : Society for Cardiovascular Magnetic Resonance (SCMR): Board of Trustees Task Force on Standardized Post-Processing. J Cardiovasc Magn Reson 22:19

4 Gwet KL (2008) Computing inter-rater reliability and its variance in the presence of high agreement. Br J Math Stat Psychol 61:29-48

5 Liu CY, Wieben O, Brittain JH, Reeder SB (2008) Improved delayed enhanced myocardial imaging with T2-Prep inversion recovery magnetization preparation. J Magn Reson Imaging 28:1280-1286

6 Peel SA, Morton G, Chiribiri A, Schuster A, Nagel E, Botnar RM (2012) Dual inversion-recovery mr imaging sequence for reduced blood signal on late gadolinium-enhanced images of myocardial scar. Radiology 264:242-249

7 Muscogiuri G, Rehwald WG, Schoepf UJ et al (2017) T(Rho) and magnetization transfer and INvErsion recovery (TRAMINER)-prepared imaging: A novel contrast-enhanced flow-independent dark-blood technique for the evaluation of myocardial late gadolinium enhancement in patients with myocardial infarction. J Magn Reson Imaging 45:1429-1437

8 Kellman P, Xue H, Olivieri LJ et al (2016) Dark blood late enhancement imaging. J Cardiovasc Magn Reson 18:77

Supplementary figure legends:

Supplementary figure 1:

11 Patients with STEMI were examined twice with the “bright-blood”- LGE sequence with different time delays between the acquisitions (median delay 7.0 min (IQR: 5.8-8.4 min). No statistically significant difference between both acquisition was found for DI between (a) scar and normal myocardium (p=0.814) and between (b) scar and left ventricular blood (p=0.305). Furthermore, no significant correlation between DI and the time after CA injection was found for (c) DI between scar and normal myocardium (r=0.048, p=0.833) and (d) DI between scar and left ventricular blood (r=0.163, p=0.632).

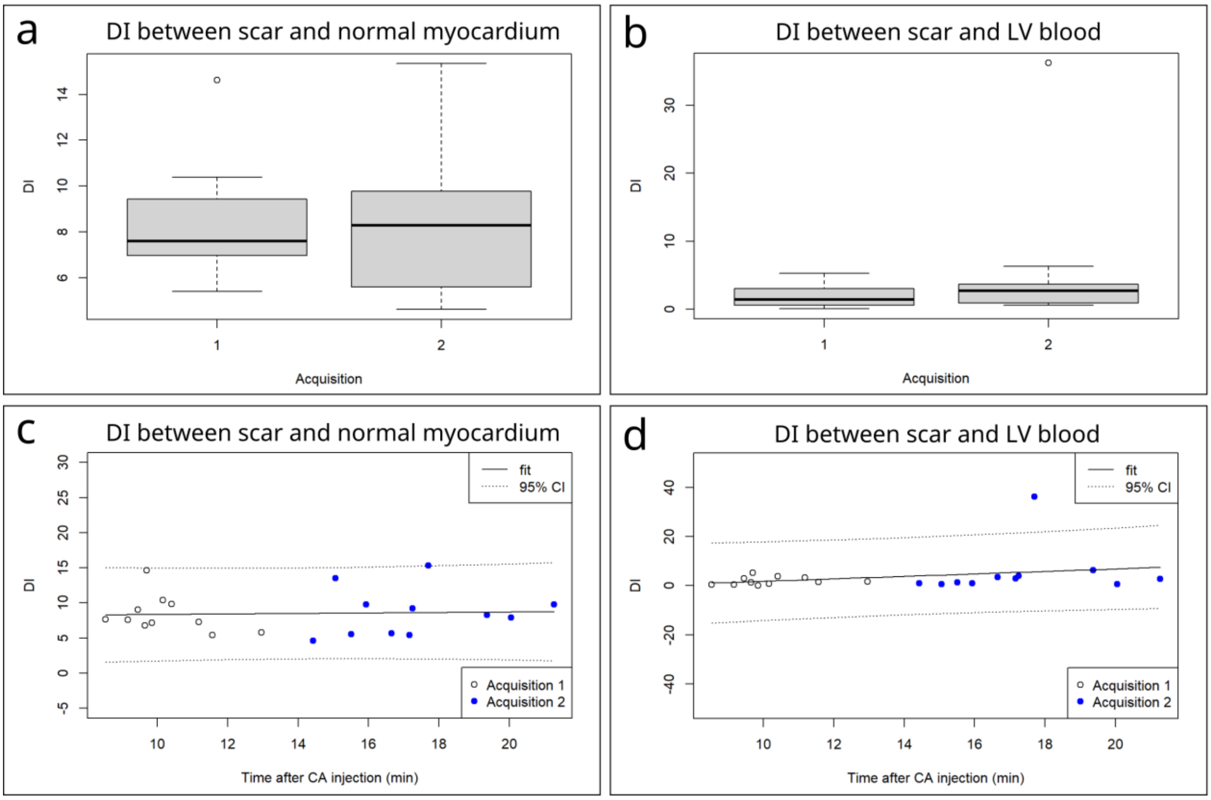

Supplementary figure 2:

11 Patients with STEMI were examined twice with the “bright-blood”- LGE sequence with different time delays between the acquisitions (median delay 7.0 min (IQR: 5.8-8.4 min). No significant difference (a) in scar volumes between both measurements (p=0.434) and no correlation (b) between the difference of infarct volume (FWHM method) of both measurements and the time delay was observed (r=0.47, p=0.140).

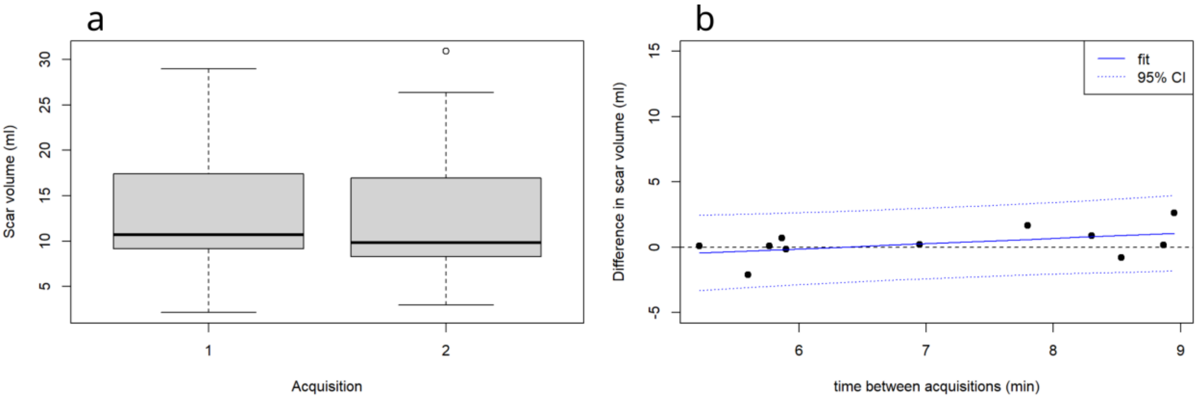

Supplementary figure 3:

Dependence of DI on the time delay after CA injection for „bright blood“ LGE (black open circles) and MT-FIDDLE (blue filled circles). (a) DI between scar and normal myocardium. (b) DI between scar and LV blood. Although a weak correlation was found for MT-FIDDLE for DI between scar and normal myocardium ANCOVA did not show a significant difference in the slopes of the regression lines (scar vs. normal myocardium: p=0.956; scar vs. LV blood: p=0.811; normal myocardium vs. LV blood: p=0.733). For all cases, ANCOVA showed a significant effect between „bright blood“ LGE and MT-FIDDLE (p < 0.001). This allows the conclusion that after adjustment for the possible influence of time delay the observed differences in DI between „bright blood“ LGE and MT-FIDDLE are significant even after adjustment for a possible influence of time delay.

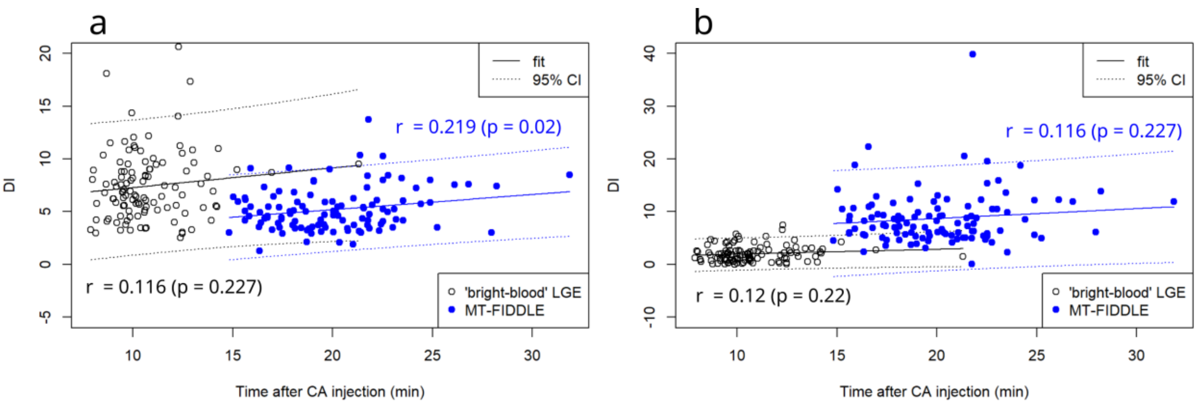
a
b
fit
95% CI
r = 0.219 (p = 0.02)
r = 0.116 (p = 0.227)
r = 0.116 (p = 0.227)
r = 0.12 (p = 0.22)
'bright-blood' LGE
MT-FIDDLE
DI
Time after CA injection (min)
